# Feasibility of personalized primary prevention (PPP) in osteoporotic fracture management

**DOI:** 10.1186/1878-5085-5-S1-A150

**Published:** 2014-02-11

**Authors:** Istvan Marton

**Affiliations:** 1 Quintess, Budapest, Hungary

## 

Age related chronic health conditions generate sever socio-economic problems in the developed countries, especially in the EU [1]. The significance and feasibility of prevention became evident, but no sound information was available to demonstrate the effectiveness of these measures [2]. Most programs running have short duration. An osteoporosis prevention program was initiated more than 20 years ago in Hungary and follow up information became accessible by now. National wide statistical figures gave solid evidence of the feasibility of PPP.

**Fig. 1:**
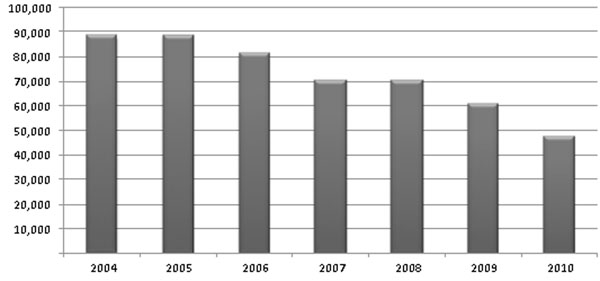
Total number of bone fractures in Hungary (population 10M) by year. Prevention initiated in 1990, significant reduction observed from 2006 onward – despite of aging population, increased life span

**Fig. 2:**
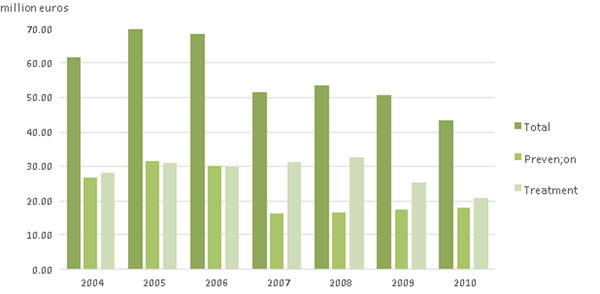
Osteoporosis related Social Security expenditure between years 2004 and 2010. Treatment cost reduced despite of inflation.

The only country within the European Union was Hungary, where – with our active ontribution - a 20 years prevention campaign to reduce osteoporotic fractures and Social security expenses - resulted in an approved positive outcome, indicated by the decreasing overall hip fracture incidence, and related expenses despite of the increased number of over 60 years age population and extended life expectancy (1990-2012) and inflation [3]. All those measures, including beneficial effects of vitamin D, are presented here. Data supported evidences of the significance of centralized data collection, and education of specialist working on integrative medical approach.
